# The Glass is Half-Full: Overestimating the Quality of a Novel Environment is Advantageous

**DOI:** 10.1371/journal.pone.0034578

**Published:** 2012-04-03

**Authors:** Oded Berger-Tal, Tal Avgar

**Affiliations:** 1 Mitrani Department of Desert Ecology, Jacob Blaustein Institutes for Desert Research, Ben-Gurion University of the Negev, Beer-Sheva, Israel; 2 Department of Integrative Biology, University of Guelph, Guelph, Ontario, Canada; University of Bristol, United Kingdom

## Abstract

According to optimal foraging theory, foraging decisions are based on the forager's current estimate of the quality of its environment. However, in a novel environment, a forager does not possess information regarding the quality of the environment, and may make a decision based on a biased estimate. We show, using a simple simulation model, that when facing uncertainty in heterogeneous environments it is better to overestimate the quality of the environment (to be an “optimist”) than underestimate it, as optimistic animals learn the true value of the environment faster due to higher exploration rate. Moreover, we show that when the animal has the capacity to remember the location and quality of resource patches, having a positively biased estimate of the environment leads to higher fitness gains than having an unbiased estimate, due to the benefits of exploration. Our study demonstrates how a simple model of foraging with incomplete information, derived directly from optimal foraging theory, can produce well documented complex space-use patterns of exploring animals.

## Introduction

For animals facing novel environments (due to dispersal or translocation) gaining information about the environment is critical, as experienced (i.e. informed) animals succeed better than animals without the relevant experience in almost all aspects of their life cycle. Such individuals are more successful at reproducing [1], [2], they are better foragers [3], [4], and they show enhanced anti-predatory behavior [5]. Thus, acquiring (and in a changing environment, also updating) information is a vital function affecting individual fitness.

Theory suggests that animals facing an unknown environment should perform exploratory movement behaviors aimed at constructing a spatial representation of the new environment in order to allow for efficient resource utilization, predator avoidance, mate location and so forth [6], [7], [8]. Exploratory behavior can be defined as the gathering of information about aspects of the environment that does not necessarily satisfy immediate needs [9]. Despite the obvious long-term advantages of exploratory behavior, it may incur high costs on the exploring animal. These costs include elevated risk of predation due to higher exposure and lower vigilance while engaged in exploring [10], [11], and increased energetic costs due to energy requirements and missed foraging opportunities [12]. Thus, animals are expected to face a trade-off between their need to learn their environment and their need to exploit the already familiar resources (the exploration-exploitation trade-off, [13]).

Once animals become familiar with their surroundings, they often restrict their foraging to a limited area that is commonly called the home range [14], [15]. This transition from an exploratory stage to foraging within a confined and familiar landscape calls for notable changes in the movement behavior of the dispersing animals [16], [17]. In environments that are composed of renewable resource patches, trap-lining, defined as repeated visitation to a series of resource patches in a predictable order, is usually the most beneficial foraging strategy [18]. Thus, dispersed or translocated animals are expected to exhibit dynamical and complex space-use patterns, shifting from exploratory movement to home range establishment and, in many cases, trap-lining. Indeed, such patterns have been reported in the wild for a wide range of species (e.g, [19]–[21]). Here, we demonstrate that these seemingly complex patterns can arise from a simple model of foraging with incomplete information, derived directly from optimal foraging theory.

According to Charnov's seminal marginal value theorem [22], optimal foragers maximize their long-term intake rate by leaving patches when the intake rate of resources in the patch falls to a rate that equals the long-term average intake rate in the environment. The theorem however, is based on several unrealistic assumptions such as a deterministic environment and a perfectly informed forager [23], [24]. As in reality environments are stochastic and real foragers do not possess perfect information about them, optimal foragers are expected to behave in an approximately Bayesian manner (i.e., to update their estimate of the environment as they forage, [24]–[26]). At any rate, an optimal forager's decisions (e.g., where to forage and for how long) are based on its current estimate of the quality of the environment. However, in a novel environment, a forager does not possess precise information regarding the quality of the environment, and may make a decision based on a biased estimate (i.e., either an overestimation or an underestimation of the true value). This is not necessarily a bad thing as several studies have demonstrated that biased behaviors in the face of uncertainty can become adaptive and evolve due to trade-offs between short-term and long term payoffs [27]–[32]. The exploration-exploitation trade-off represents such a trade-off and might therefore call for a biased behavior as an adaptive lifetime strategy.

We argue that in the face of a novel environment, having a biased initial estimate of the environment is in many cases an adaptive strategy. Specifically, we argue that for animals that can move with relative ease between patches (e.g., ungulates or rodents), having a positively biased estimate of the environment (being an “optimist”) promotes exploration and is consequently a more successful strategy than having a negatively biased estimate (being a “pessimist”). This is due to the fact that an optimistic forager will underestimate the relative value of the patches it encounters (compared to the expected environment), and will more readily leave them. Since optimistic foragers explore more than pessimistic foragers, they will also be quicker in learning the true value of the environment and improve their decision making more rapidly. We further argue that when the forager has the ability to remember the location and quality of resource patches, being an optimist can be a better strategy than having an unbiased (“correct”) estimate of the environment, as an optimistic forager may encounter high quality patches during its explorations, converging on a trap-line of a higher quality than the average of the environment.

To validate our predictions, we constructed a simple spatially implicit model simulating foraging with incomplete information in a novel heterogeneous landscape of renewable resource patches.

## Methods

We consider an animal foraging in a novel environment following the simple rules of Charnov's marginal value theorem [22] – the forager leaves a patch once its intake rate drops below the expected intake rate elsewhere. We look at three different types of foragers according to their learning and spatial memory capabilities. We define learning as the ability of the forager to update its estimate of the environment's mean quality as it encounters new patches, and spatial memory as the ability of the forager to store the attributes of each visited patch and return to it at a later stage. While memory is an integral part of any learning mechanism, we separate spatial memory from learning. A forager without spatial memory can still learn (i.e., update its estimate of the environment as it encounters new patches), but it cannot assign the newly learned value to a particular location and is unable to return to any of the patches it previously visited. Similarly, a forager can remember the location and quality of each patch it visited but not update its expectation regarding the quality of a novel patch (i.e., not learn). We investigated the performances of a forager that can learn but cannot remember, a forager that can remember but cannot learn, and finally, a forager that can both learn and remember.

### Model overview

Our model simulates the foraging patterns of a consumer feeding in, and moving among, discrete, heterogeneous and renewable resource patches following the general approach of [33]–[34]. The time required to consume a single *food unit* (the term is used here to denote a mouthful, a bite, or a single resource item) is calculated based on patch density (the number of available food units in the patch), according to a type II functional response [35]. Search rate and handling time are kept constant throughout all simulations (0.01 and 1 respectively; a preliminary sensitivity analysis revealed little qualitative effects on the simulation results). The forager is assumed to instantly know the quality of the patch upon encountering it. After the consumption of each food unit, a decision is made of whether to stay in the current patch or move to another. This decision is based on the assumption that if patch departure is the best alternative, the current patch density is the optimal giving-up density (GUD, [36]–[37]). The expected intake rate in the next patch is calculated as the number of food units available in that patch above the current GUD, divided by the time it should take to consume them (based on the functional response and the expected travel time). If this expected intake rate exceeds the expected immediate intake rate at the current patch (the inverse of the time required to consume the next food unit), the consumer will shift to a new patch where it would consume the next food unit. Otherwise, the consumer will consume the next food unit in the current patch. This sequence of events is repeated until some predefined number of food units has been consumed. We assume that the rate of energy expenditure is constant, regardless of whether the animal is foraging or moving between patches. This assumption is reasonable as many animals continuously move (and thus spend energy) while they forage.

The environment is heterogeneous with respect to two properties. The maximum quality of each of the resource patches (i.e., the quality in the absence of any consumption) is drawn from a Poisson distribution whose average represents the mean landscape quality. The traveling time between patches is drawn from an exponential distribution with an average that represents the mean traveling time in this landscape. In the results presented here, both the mean landscape quality and the mean traveling time were kept constant (100 and 10 respectively) after an extensive sensitivity analysis revealed that changes in these parameters did not qualitatively change the results.

We define a forager as *optimistic* or *pessimistic* according to its estimate of the environment's quality. A forager is considered an optimist if its initial estimate of the environment is positively biased (i.e. higher than the true average quality of the environment). In a similar fashion, a forager is considered a pessimist if its initial estimate of the environment is negatively biased. The model focuses on the estimates of patch quality and assumes the estimate of the mean traveling time is unbiased as the two aspects have similar quantitative effects with no non-additive interactions. When the forager has no learning capabilities its estimate of the environment remains constant throughout the simulation. Note that our definitions of optimism and pessimism differ from those found in ref. [31] as we do not refer to reproduction but rather to the way an animal assesses its environment. However, just as in ref. [31], our definitions are purely mechanistic and make no assumptions regarding the mental state of the forager.

### Learning

In our model, a forager with learning capabilities updates its estimate of the environment's quality every time it visits a new patch as a weighted mean of its past estimate and the quality of the current patch. We used the linear operator rule [38]–[39]:
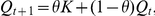
where *Q_t_* is the estimate of the environment's quality at time *t*, *K* is the quality of the current patch, and *θ* is the weighing factor ranging from 0 to 1. A large *θ* results in a very rapid update of the forager's estimate of the environment with every new patch it encounters, while a *θ* of zero result in an estimate that always stays constant (i.e., there is no learning). In the results presented here, a learning forager had a *θ* of 0.01. The linear operator rule has been shown to perform well relative to other learning rules [40], and often nearly as well as computationally demanding Bayesian learning strategies [41]. However, like any other rule that is Bayesian in nature, it is dependent on the animal having a prior expectation of the quality of the environment (i.e. the forager's initial estimate of the environment *Q_t = 0_* must have some value). We refer to a forager as optimistic or pessimistic according to its initial estimate of the environment as explained above.

### Spatial memory

A forager with spatial memory stores the location and quality of every patch it encounters in its memory, and can decide to return to these patches at a later time. Thus, a forager with spatial memory faces three options after consuming every food unit – to continue feeding in the current patch, to leave the patch and return to a previously visited patch, or to travel to a new patch. As before, the decision is made according to the best expected long-term intake rate. Resource patches in our simulation regenerate according to a logistic growth model [42] where patch quality at time *t*, *k_t_*, is a function of the maximum patch quality, *K*, the patch's quality *Δt* time units ago, *k_t−Δt_*, and the maximum growth rate, *r* ( = 0.001):
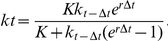
Hence, the rate of patch re-growth diminishes as the patch approaches its maximum quality. We assume that the forager has perfect knowledge of the rate of patch renewal and therefore knows the current quality of each previously visited patch.

### Fitness currency

The simulation ends once a forager consumes a fixed amount of food units. Depending on their patch departure decisions, different foragers consume the same fixed amount of food at different rates. We considered foragers with high long-term intake rate (i.e., foragers who consumed the fixed amount of food rapidly) as foragers with higher fitness than foragers who took longer to consume the same amount of food. The use of long-term intake rate is a common proxy of fitness, especially in an optimal foraging framework [43]. As the relative performance of different foraging strategies may vary with the time-frame available for foraging, we consider three different time-frames: short, intermediate and long, by varying the amount of food that the forager needs to consume (100, 1000, and 10,000 food units respectively).

Appendix S1 gives a description of the parameters used in the simulations. We conducted a sensitivity analysis to verify that our results are not qualitatively affected by the values of any of the fixed parameters in our model. In our sensitivity analysis we repeated all simulations, each time changing one of the fixed parameters by either reducing or increasing its value by one order of magnitude (except for *θ* where we only increased its value, as one of our treatments includes reducing it to 0). In all cases the qualitative results of the model did not change as a function of variations in these parameters.

A MATLAB 7.6.0 (MathWorks, Natick, Massachusetts) simulation codes are given in Appendix S2 (simulation without spatial memory) and Appendix S3 (simulation with spatial memory). Each unique parameters combination was used to simulate 100 replicates (differing due to the stochastic nature of the landscape).

## Results

### Foragers with learning capabilities but no spatial memory

**Figure 1:**
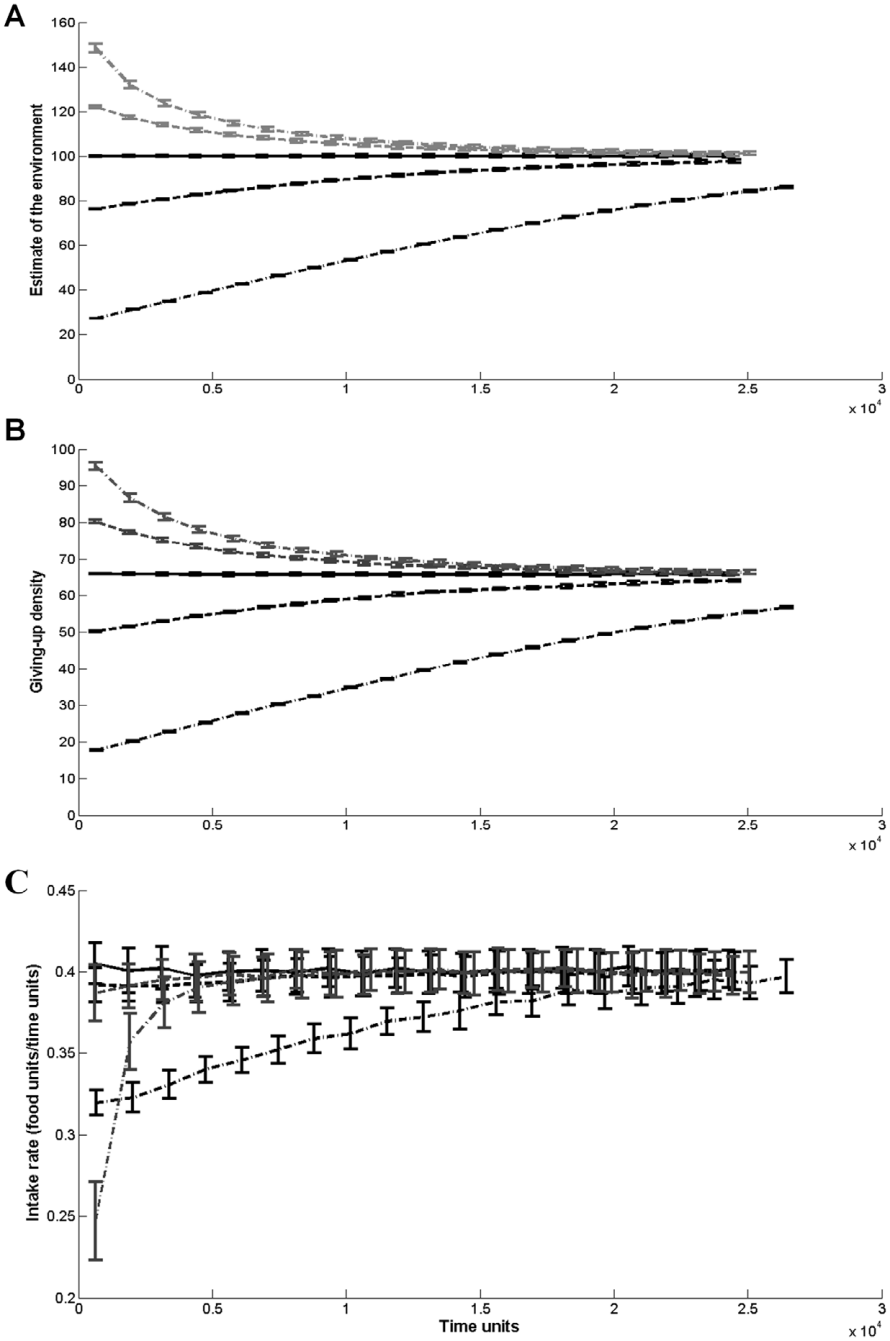
The estimate of the environment, giving-up density, and intake rate (panels a-c respectively) as a function of time, for foragers with learning capabilities but no spatial memory. The lines' color and shape represent different foragers making decisions according to different estimates of the environment quality. The black solid line represents foragers making their decision according to an estimate of 100 food units, which is equal to the true mean value of the environment quality. The dashed black line represents mild pessimists making their decisions according to an estimate of 75 food units - a 25% negative bias. The dashed-dotted black line represents extreme pessimists with an estimate of 25 food units – a 75% negative bias. The dashed and dashed-dotted gray lines represent mild and extreme optimists with a 25% and 75% positive bias respectively (i.e., estimates of 125 and 175 food units). In all cases the lines represent the average of 100 simulation runs, each terminated after the consumption of 10,000 food units. Error bars represent one standard deviation.

When the forager is able to learn but has no spatial memory, the best initial strategy was to know the true value of the environment's quality (Fig. 1). Nonetheless, there was a difference between the performances of foragers with an optimistic strategy and foragers with a pessimistic one. All foragers updated their estimate of their environment based on every new resource patch they encountered. However, due to their higher exploration rate (the number of arrivals to new patches per time unit), the rate at which the optimistic foragers updated their estimate of the environment was much higher than that of the pessimistic foragers (Figure 1a), resulting in a more rapid convergence to the optimal GUD (i.e., the GUD of perfectly informed foragers according to the marginal value theorem; Fig. 1b). Foragers with mild biases in their estimate of the environment had practically the same intake rate (amount of food consumed per time unit) as a perfectly informed forager. The initial intake rate of foragers with extreme biases was much lower than the intake rate of the perfectly informed forager, with the extreme optimistic strategy being the worst at first. However, the extremely optimistic forager was quick to improve its intake rate and equalize it to the optimal one, while the extremely pessimistic forager had a sub-optimal intake rate for a much longer period of time (Fig. 1c).

### Foragers with spatial memory but no learning capabilities

**Figure 2:**
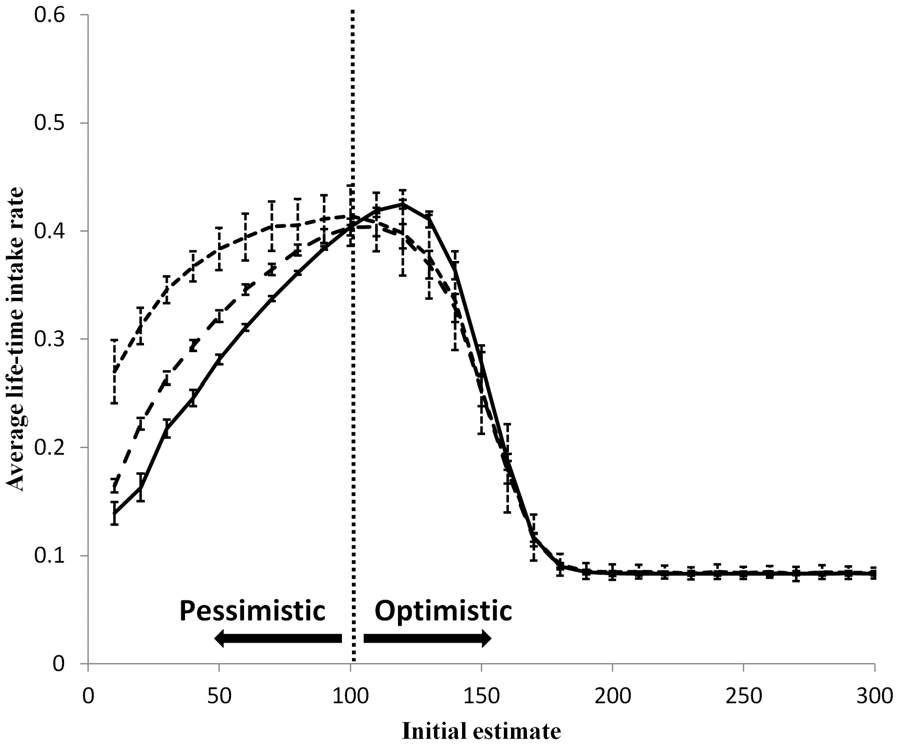
The average life-time intake rate (the intake rate average over the whole period of the simulation) as a function of the initial estimate of the environment quality for foragers with memory but without learning capabilities. The solid line represents a forager with a long time-frame of foraging (i.e., a simulation which ended after the forager consumed 10,000 food units). The dashed line represents a forager with an intermediate time-frame (1,000 food units), and the dotted line represents a forager with a short time-frame (100 food units). The dotted vertical line indicates on the average quality of the environment. Error bars represent one standard deviation.

The average life-time intake rate of remembering-yet-not-learning foragers was influenced both by their estimate of the environment (which remained constant throughout the simulation) and by the time-frame (the predefined resource quantity to be consumed during the simulation). Foragers whose time-frame of foraging was long (10000 food units) had the highest life-time intake rate when they were mildly optimistic (i.e., when their estimate of the environment was 20% higher than the true average of the environment). Foragers with an intermediate time-frame of foraging (1000 food units) showed a similar but weaker trend, and foragers whose time-frame was short (100 food units), had the highest intake rate when their estimation of the environment equaled its true value (Fig. 2).

**Figure 3:**
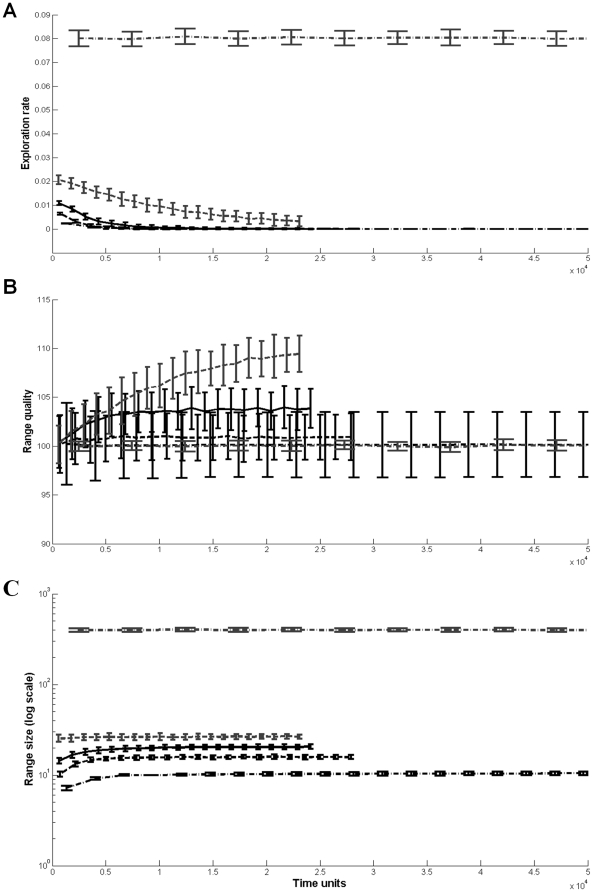
The exploration rate, average range quality, and range size (panels a–c respectively) as a function of time, for foragers with spatial memory but without learning capabilities. The different lines represent foragers with different initial estimates of the environment, as detailed in the caption for figure 1. Error bars represent one standard deviation.

Initial exploration rates (the number of arrivals to new patches per time unit) reflected the direction and magnitude of the foragers' initial estimate bias. Despite the fact that foragers did not update their estimate of the environment, the exploration rate of all foragers but the extreme optimist declined with time until the foragers performed no exploration (Fig. 3a). This decline in exploration rate was the result of foraging in a subset of the landscape that was at least as valuable as the forager's estimate (the extreme optimist continued exploring as no subset could match its high expectations). This happened quickly for pessimistic foragers, and more slowly for the mild optimistic forager who explored until the average value of the patches it visited increased to its estimation of the environment. These trends are reflected in the temporal dynamics of the foragers' range quality (the average quality of visited patches within each of 20 equal time bins; Fig. 3b). Finally, for all the foragers, the range size (the number of unique patches visited within each of 20 equal time bins) stabilized and became constant over time with significantly higher values for the extreme optimist (Fig. 3c).

### Foragers with both spatial memory and learning capabilities

**Figure 4:**
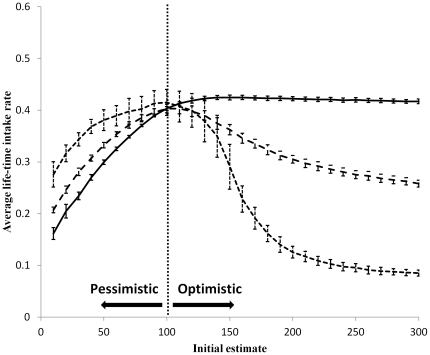
The average life-time intake rate (the intake rate average over the whole period of the simulation) as a function of the initial estimate of the environment quality for foragers with both spatial memory and learning capabilities. The solid line represents a forager with a long time-frame of foraging (i.e., a simulation which ended after the forager consumed 10,000 food units). The dashed line represents a forager with an intermediate time-frame (1,000 food units), and the dotted line represents a forager with a short time-frame (100 food units). The dotted vertical line indicates on the average quality of the environment. Error bars represent one standard deviation.

**Figure 5:**
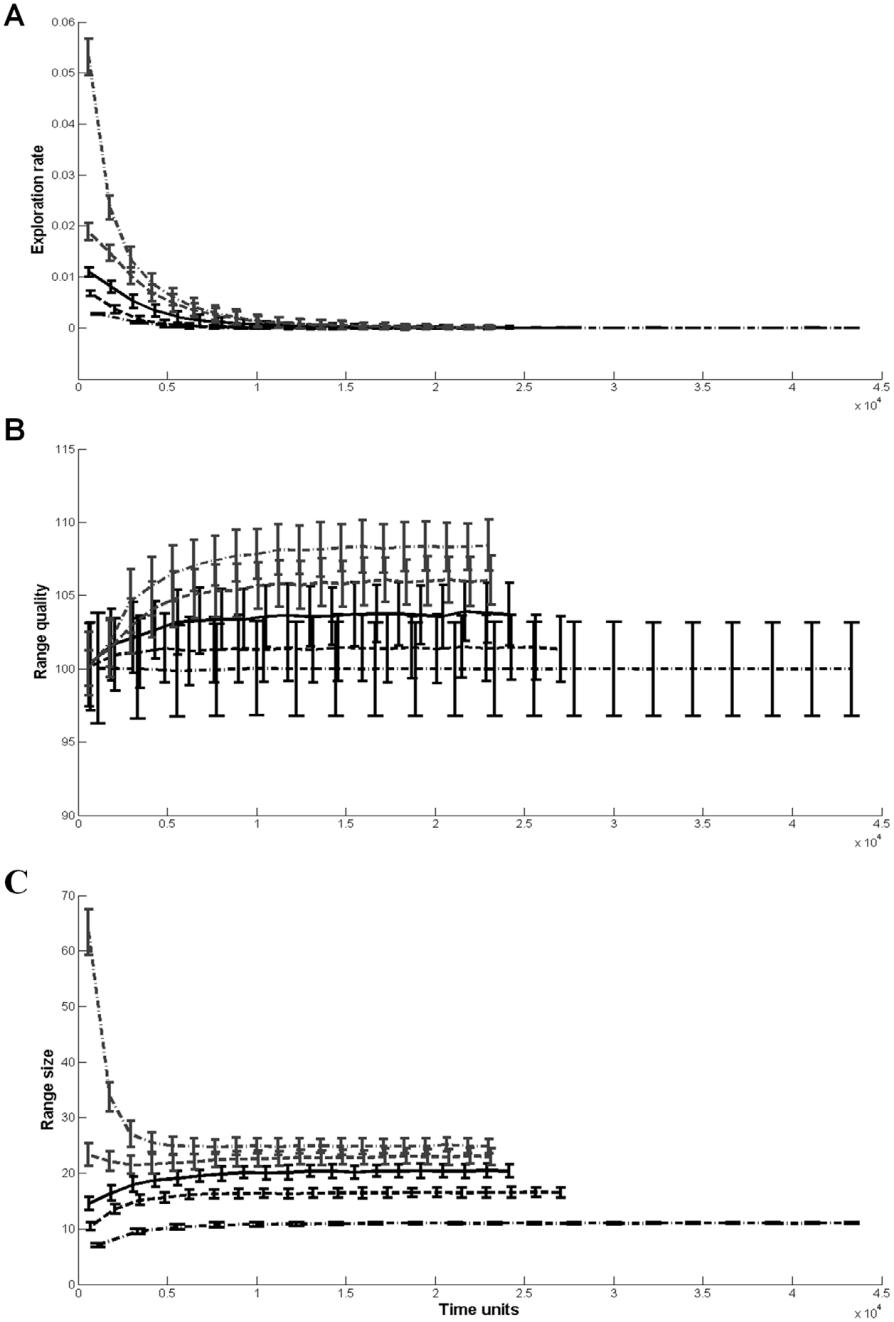
The exploration rate, average range quality; and range size (panels a–c respectively) as a function of time, for foragers with both spatial memory and learning capabilities. The different lines represent foragers with different initial estimates of the environment, as detailed in the caption for figure 1. Error bars represent one standard deviation.

Learning and memorizing foragers with a long time-frame of foraging had the highest life-time intake rate when they were optimistic, regardless of how optimistic they were (Fig. 4). As the time-frame of foraging decreased, the price for extreme optimism increased. Foragers with a short time-frame exhibited the same life-time intake rate curve as forgers with a short time-frame and no learning capabilities (Fig. 2). All foragers decreased their exploration rate with time until they stopped exploring, converging to a stable range with a stable average value (Fig. 5).

## Discussion

We have shown that in many cases having a positively biased estimate of the environment's quality (i.e., being an “optimist”) outperforms pessimism in a novel environment. Moreover, when an animal can remember and therefore return to previously visited resource patches, optimism may perform even better than having an unbiased estimate of the environment.

One of the better known concepts in optimal foraging theory is Charnov's marginal value theorem [22] that considers when a forager should leave a patch of resources. Criticism of the theorem's assumptions regarding the deterministic nature of the resource patches led to the rise of Bayesian foraging theory [23]–[25]. Bayesian foragers decide when to leave a resource patch based on the weighted average of a prior estimate of patch qualities and sampling information from the current patch [44]. This strategy is more successful in stochastic environments than classical optimal foraging. The main criticism of the Bayesian approach is over the source of the prior estimate of the environment quality [45]–[46], which is supposedly gained by past experience or through evolution by natural selection [24]. Our results demonstrate that a positively biased prior estimate of the environment converges to the true estimate of the environment much faster than a negatively biased estimate of the same magnitude. This is due to the fact that an optimistic forager's overestimation of the environment is motivating it to leave resource patches and explore. The high exploration rate allows for a fast update of the estimate of the environment compared to a pessimistic forager's much slower update rate. This is true regardless of the travel distance between the patches (see methods). Thus, when an animal with learning capabilities faces a novel environment and is required to “guess” its quality in order to make foraging decisions, overshooting is always a better strategy than undershooting, making optimism an adaptive strategy that can evolve by natural selection.

We consider a forager that overestimates the quality of the environment as an optimist. However, it's worth noting that what we regard as optimism about the environment can also be regarded as pessimism towards the quality of the current patch. Optimism regarding the quality of the environment will promote exploration while optimism regarding the quality of the current patch will have the opposite effect.

A forager with spatial memory capabilities is able to re-visit high quality patches, creating a sub-group of patches of a higher average quality than the environment's average. As long as the forager's positive bias is relatively small, the increase in the average quality of the forager's memorized environment can reduce its exploration rate without the need for a learning mechanism. Instead of adjusting its perceived value of the environment, the forager is focusing its activity in a high quality subset of its environment. Thus, memory (or spatial learning) can serve as an alternative mechanism to learning for decreasing exploration with time. In the case of extreme optimists, learning capabilities are a necessity in order to avoid the high costs of constant exploration (as the forager can never converge to a range that has a quality as high as it expectation; Fig. 2, 3).

While the notion of animals using optimism to promote exploration in order to improve the quality of their future home range is novel, the use of optimism to promote exploration is widely used in the fields of machine learning, artificial intelligence, and neural network research [32]. Just like foragers, machines that learn need to balance between exploration (of actions whose outcomes are still not well known) and exploitation (of actions with known positive outcomes). By having an optimistic initial value, a learning apparatus is encouraged to explore different actions, thereby gaining valuable information on the distribution of their outcomes which can improve their future performance [32].

One of the characteristics of the exploration-exploitation trade-off is that there is, to some extent, a temporal partition between the costs and the benefits of exploration [13] - a forager in a novel environment is expected to first pay a cost for high exploration rates which in later stages will be superseded by the benefits of information. Thus, the forager's relevant time frame or life expectancy should have a strong influence on the advantages of exploratory behavior. Indeed, our results show that as the relevant time frame becomes shorter, the benefit from exploration decreases, and the animal should be less optimistic. This follows closely the theoretical and empirical findings ref. [47] who found that female *Anaphes victus* parasitoids remained for a longer time on host patches as they approached the end of their life (i.e., the females reduced their exploration rate as the time horizon for foraging diminished).

Animals facing novel environments exhibit dynamical and complex space-use patterns, shifting from exploratory movement to home range establishment [19]–[21]. We showed that these seemingly complex patterns can arise from a simple model of foraging with incomplete information, derived directly from optimal foraging theory. Optimistic animals show a high rate of exploratory movements which declines with time. This decline is not enforced on the forager, but rather an emergent property of the model – as the forager encounters new patches, it reduces its estimate of the environment, which in turn decreases its exploration rate. In order to facilitate the establishment of a home range, a forager requires memory capabilities [15]. In our model, memory-enabled foragers with learning capabilities reduced their exploration rate to zero after a certain amount of time (Fig. 5a), indicating the establishment of a stable home range. With the exception of ref. [15], movement models that lead to the emergence of a home range behavior out of potentially unrestricted movement paths have only been demonstrated for territorial species or central-place foragers [48]. Ref. [15] presented a model based on correlated random walk in which the use of a two-part memory system by a foraging animal could lead to an establishment of a stable home range. We show that by simply allowing an optimal forager with incomplete information to update its estimate of the environment while foraging, stable home range behavior can emerge after a period of nomadic exploration. Moreover, if the initial estimate bias is small enough, and as long as the forager has spatial memory, stable home range behavior can emerge from optimal foraging patch leaving rules even without the need for a learning mechanism.

In our simulation, the number of patches a forager with spatial memory visited and their average quality became stable over time, which strongly suggests, albeit indirectly, that it was trap-lining (but see ref. [49] on the difficulties of statistically identifying trap-lining). One of the costs of complete trap-lining (i.e., having an invariable foraging route between a set of known patches) is that if the value of the patches changes with time, the forager might get ‘stuck’ in an inefficient foraging route. Ref. [18] suggested that foragers should switch between complete trap-lining and ‘sample and shift’ trap-lining (i.e., when the forager encounters a less rewarding patch in its trap-line, it might leave it and go exploring for an alternative patch). Although not the focus of the current investigation, such a strategy is likely to emerge in our model as a result of degradation in the quality of patches that are part of the forager's trap-line. The forager would then have a higher estimate of the environment's average than the current average in these patches, motivating it to explore new patches and update its trap-line, thus performing a ‘sample and shift’ trap-line. The emergence of such complex temporal dynamics from a simple set of foraging and learning rules may help identify possible behavioral response to environmental changes and should be investigated in future research.

Two major assumptions of our model require further discussion. First, the costs of exploration in our model are solely due to the fact that the exploring animal foregoes resource exploitation in order to travel to a new patch (which may or may not be a better patch). The underlying assumption of this approach is that the energetic cost of moving between patches is equivalent to the energetic cost of foraging in a patch. There are obviously instances at which this assumption fails due to high energetic costs of locomotion or to increased risk of predation while on the move [36], [50]. However, as long as these additional costs increase linearly with time spent traveling (and the forager is fully aware of them), their inclusion should have the same effect as increasing the distances between patches. While this has a quantitative effect on our results (i.e., all intake rates are suppressed and a longer time-frame is required for optimism to outperform perfect information), it does not have any qualitative effect on our conclusions. The second assumption is the focus on the optimal behavior of a single forager while in reality, foragers are rarely alone. When in a group, foragers can gain information by following other individuals (social information, [51]). This may lead to producers-scroungers dynamics [52] in which some individuals specialize in exploring while others specialize in following explorers. Alternatively, multiple foragers may compete by exploitation, thus destabilizing any individual trap-line. At any rate, for non-solitary foragers, the advantages of optimism may be frequency dependent.

We have demonstrated how movement and food consumption patterns might emerge and evolve as a result of the interaction between animals' memory and learning abilities and their prior expectations from a novel environment. These ideas could and should be put to empirical tests using captive or relocated animals. By first conditioning individuals to an environment of certain mean quality and then introducing these individuals to a novel environment characterized by a similar or a different mean quality, one might be able to directly test the ideas suggested here. Furthermore, additional theoretical research is needed to test the validity of our results in explicit space and with different levels of spatial and temporal variability in resource quality (such as when the resources are clumped, [44]). At any rate, this work adds to recent advances (e.g., [53]) linking optimal foraging theory and the newly emerging movement ecology paradigm [54]. A link that should help promote a deeper cognitive-adaptive understanding of animal space use patterns.

## Supporting Information

Appendix S1 **A description of the different parameters used in the model.**
(PDF)

Appendix S2 **Matlab code for the foraging simulation without memory.**
(PDF)

Appendix S3 **Matlab code for the foraging simulation with memory.**
(PDF)
