# Supplementary material for: The Glass is Half-Full: Overestimating the Quality of a Novel Environment is Advantageous
Source: PLoS One. 2012 Apr 3;7(4):e34578. doi: 10.1371/journal.pone.0034578 (PMC3317990; doi:10.1371/journal.pone.0034578)
Supplement: Appendix S1 — A description of the different parameters used in the model. (PDF) [file pone.0034578.s001.pdf]

**parameter**	**description**	**value/s**
bites	the number of food units to be consumed throughout the simulation	100-100,000 [food-units]
mean patch quality	the mean Poisson distributed number of food units available in a new patch; the quality of landscape.	100 [food-units/patch]
presumed mean patch quality	the initial belief of the forager regarding the landscape quality; values >100 correspond to 'optimism' while values <100 correspond to 'pessimism'.	25-175 [food-units/patch]
mean travel time	the mean exponentially distributed travel time across the landscape; the average time required to locate a new patch.	10 [time-units]
food growth rate	the logistic renewal rate of food patches	0.001 [time-units^{-1}]
handling time	the time required to process a single food unit	1 [time-units]
search rate	the fraction of a patch covered by the forager during each time spent searching for food.	0.01 [patch/time-units]
θ	the linear operator determining the rate of information update. If θ =0 the forager never learns and thus maintain its initial beliefs throughout the simulation; if θ=1 the forager would believe the mean patch quality is equal to those characterising its current location.	0.01
