# Supplementary material for: The Glass is Half-Full: Overestimating the Quality of a Novel Environment is Advantageous
Source: PLoS One. 2012 Apr 3;7(4):e34578. doi: 10.1371/journal.pone.0034578 (PMC3317990; doi:10.1371/journal.pone.0034578)
Supplement: Appendix S2 — Matlab code for the foraging simulation without memory. (PDF) [file pone.0034578.s002.pdf]

```
clear;

%% parameterization
%the number of 'bites' or food items to be consumed by the end of the simulation.
bites=10000;

%the mean of the Poisson distributed number of bites available in a new patch
mean_maximum_patch_quality=100;
%the prior - the forager's initial belief regarding the mean maximum patch quality. A high value
corresponds to 'optimism' while a low value corresponds to 'pessimism'
presumed_mean_maximum_patch_quality=mean_maximum_patch_quality;

%the mean of the exponentially distributed time required to locate a new patch
mean_exploration_time=10;
%the prior - the forager's initial belief regarding the mean exploration time. A low value corresponds to
'optimism' while a high value corresponds to 'pessimism'
presumed_exploration_time=mean_exploration_time;

%the parameters of the functional response
handling_time=1;
search_rate=1/(handling_time*mean_maximum_patch_quality);%at the mean maximum patch quality
the intake rate is half of the maximum intake rate.

%theta is the linear operator determining the rate of information update. If theta = 0 the forager never
learns and thus maintain its initial beliefs throughout the simulation; if theta = 1 the forager would
believe the mean maximum patch quality and travel time are equal to those characterizing its current
location.
theta=0.01;

%% initialising the simulation
%these vectors store prior values, consumption times, explorations (i.e., incidence of moving into a new
patch) and giving-up densities (GUDs; the densities at which each patch was departed) through the
simulation.
prior_maximum_patch_quality=NaN(bites,1);
prior_exploration_time=NaN(bites,1);
time=NaN(bites,1);
patch_exploration=false(bites,1);
patch_GUD=NaN(bites,1);

%the simulation begins as the forager moves into a new patch
patch_exploration(1)=true;
```

```
%the current patch maximum patch quality is drawn form a Poisson distribution with a minimum value
of 1 bite.
current_maximum_patch_quality=poissrnd(mean_maximum_patch_quality-1)+1;
%the maximum patch quality prior is updated based on the quality of the current patch
prior_maximum_patch_quality(1)=(theta*current_maximum_patch_quality)+((1-
theta)*presumed_mean_maximum_patch_quality);
%the current patch exploration time is drawn form an exponential distribution with a minimum value of
1 bite.
current_exploration_time=exprnd(mean_exploration_time-1)+1;
%the exploration time prior is updated based on the time to the current patch
prior_exploration_time(1)=(theta*current_exploration_time)+((1-theta)*presumed_exploration_time);
%the time required to consume a single bite is the inverse of the type II functional response + the time
spent traveling:
time(1)=(1/(search_rate*current_maximum_patch_quality))+handling_time+current_exploration_time;
%the current patch quality is updated after the consumption of a single bite
current_patch_quality=current_maximum_patch_quality-1;

%% the following loop runs over all bites
for b=2:bites

  %% decide whether to explore
  %the expected patch quality is the number of bites the forager expects to find in a new patch based
on its current knowledge of its environment. If this expectation is lower than the current patch quality,
the forager will not explore
  expected_patch_quality=round(prior_maximum_patch_quality(b-1));
  if current_patch_quality>=expected_patch_quality
    patch_exploration(b)=false;
  else
    %otherwise, if the forager shifts to the next patch, the current patch density is the GUD and the
mean intake rate in the next patch can be calculated based on the assumption that it will have the same
GUD.
    next_patch_intake=expected_patch_quality-current_patch_quality;
    %the time the forager expects to stay in the next patch (including traveling time):
    next_patch_time=prior_exploration_time(b-1)+(next_patch_intake*handling_time)+...
      ((1/search_rate)*sum(1./(expected_patch_quality:-1:(current_patch_quality+1))));
    %the mean intake rate expected during this time:
    next_patch_intake_rate=next_patch_intake/next_patch_time;
    %the intake rate in the current patch during the next bite:

current_intake_rate=(search_rate*current_patch_quality)/(1+(search_rate*current_patch_quality*han
dling_time));
```

```
        %the forager will depart the current patch if the long term expected intake rate exceeds the current
one
        patch_exploration(b)=next_patch_intake_rate>current_intake_rate;
    end

    %% consume the next bite
    if patch_exploration(b)
        %in case the forager shifts into a new patch
        patch_GUD(b-1)=current_patch_quality;
        %the new patch maximum patch quality is drawn form a Poisson distribution with a minimum value
of 1 bite.
        current_maximum_patch_quality=poissrnd(mean_maximum_patch_quality-1)+1;
        %the maximum patch quality prior is updated based on the quality of the new patch
        prior_maximum_patch_quality(b)=(theta*current_maximum_patch_quality)+((1-
theta)*prior_maximum_patch_quality(b-1));
        %the new patch exploration time is drawn form an exponential distribution with a minimum value
of 1 bite.
        current_exploration_time=exprnd(mean_exploration_time-1)+1;
        %the exploration time prior is updated based on the time to the new patch
        prior_exploration_time(b)=(theta*current_exploration_time)+((1-theta)*prior_exploration_time(b-
1));
        %the time required to consume a single bite is the inverse of the type II functional response + the
time spent traveling:

time(b)=(1/(search_rate*current_maximum_patch_quality))+handling_time+current_exploration_time;
        %the new patch quality is updated after the consumption of a single bite
        current_patch_quality=current_maximum_patch_quality-1;

    else
        %in case the forager stays in the current patch.
        %in the absence of new information, the priors remain unchanged.
        prior_maximum_patch_quality(b)=prior_maximum_patch_quality(b-1);
        prior_exploration_time(b)=prior_exploration_time(b-1);
        %the time required to consume a single bite is the inverse of the type II functional response:
        time(b)=(1/(search_rate*current_patch_quality))+handling_time;
        %the current patch quality is updated after the consumption of a single bite
        current_patch_quality=current_patch_quality-1;
    end
    %repeat until all bites are consumed
end
```
