# Supplementary material for: The Glass is Half-Full: Overestimating the Quality of a Novel Environment is Advantageous
Source: PLoS One. 2012 Apr 3;7(4):e34578. doi: 10.1371/journal.pone.0034578 (PMC3317990; doi:10.1371/journal.pone.0034578)
Supplement: Appendix S3 — Matlab code for the foraging simulation with memory. (PDF) [file pone.0034578.s003.pdf]

```
clear;

%% parameterisation
%the number of 'bites' or food items to be consumed by the end of the simulation.
bites=10000;

%the mean of the Poisson distributed number of bites available in a new patch
mean_maximum_patch_quality=100;
%the prior - the forager's initial belief regarding the mean maximum patch quality. A high value corresponds to 'optimism' while a low value corresponds to 'pessimism'
presumed_mean_maximum_patch_quality=2*mean_maximum_patch_quality;

%the mean of the exponentially distributed time required to locate a new patch
mean_exploration_time=10;
%the prior - the forager's initial belief regarding the mean exploration time. A low value corresponds to 'optimism' while a high value corresponds to 'pessimism'
presumed_exploration_time=0.5*mean_exploration_time;

%the logistic growth rate of forage availability
growth_rate=0.001;

%the parameters of the functional response
handling_time=1;
search_rate=1/(handling_time*mean_maximum_patch_quality);%at the mean maximum patch quality the intake rate is half of the maximum intake rate.

%theta is the linear operator determining the rate of information update. If theta=0 the forager never learns and thus maintain its initial beliefs throughout the simulation; if theta=1 the forager would believe the mean maximum patch quality and travel time are equal to those characterising its current location.
theta=0.01;

%% initialising the simulation
%these vectors store prior values, consumption times, explorations (i.e., incidence of moving into a new patch), giving-up densities (GUDs; the densities at which each patch was departed) and patch ID's through the simulation.
prior_maximum_patch_quality=NaN(bites,1);
prior_exploration_time=NaN(bites,1);
time=NaN(bites,1);
patch_exploration=false(bites,1);
patch_GUD=NaN(bites,1);
```

```
patch_indices=NaN(bites,1);

%the simulation begins as the forager moves into a new patch
current_patch_index=1;
patch_exploration(1)=true;
%the current patch maximum patch quality is drawn form a Poisson distribution with a minimum value
of 2 bites.
current_maximum_patch_quality=poissrnd(mean_maximum_patch_quality-2)+2;
%this vector keeps track of the carrying capacities of all visited patches:
maximum_patch_qualities=current_maximum_patch_quality;
%the maximum patch quality prior is updated based on the quality of the current patch
prior_maximum_patch_quality(1)=(theta*current_maximum_patch_quality)+((1-
theta)*presumed_mean_maximum_patch_quality);
%the current patch exploration time is drawn form an exponential distribution with a minimum value of
1 bite.
current_exploration_time=exprnd(mean_exploration_time-1)+1;
%this vector keeps track of the exploration times of all visited patches:
exploration_times=current_exploration_time;
%the exploration time prior is updated based on the time to the current patch
prior_exploration_time(1)=(theta*current_exploration_time)+((1-theta)*presumed_exploration_time);
%the time required to consume a single bite is the inverse of the type II functional response + the time
spent traveling:
time(1)=(1/(search_rate*current_maximum_patch_quality))+handling_time+current_exploration_time;
%this vector keeps track of current patch qualities:
current_qualities=current_maximum_patch_quality-1;

%% the following loop runs over all bites
for b=2:bites

  %% the memory is updated after each bite
  %keeps track of patch identity:
  patch_indices(b-1)=current_patch_index;
  %update the current quality of all memorised patches based on a logistic growth model:
  current_qualities=(maximum_patch_qualities.*current_qualities.*exp(growth_rate.*time(b-1)))./...
    (maximum_patch_qualities+(current_qualities.*(exp(growth_rate.*time(b-1))-1)));
  current_qualities(current_patch_index)=round(current_qualities(current_patch_index));

  %% calculate the expected intake rate in the best memorised patch
  %find all memorised patches with higher quality than the current patch
  indices=find(round(current_qualities)>current_qualities(current_patch_index));
  if  any(indices)
    %if such patches exist, calculate the expected intake rate in each one:
```

```
        memorised_patch_intake_rates=NaN(1,length(indices));
        for i=1:length(indices)
            expected_quality=round(current_qualities(indices(i)));
            %calculate the expected intake rate in case of shifting to this patch and depleting it to the current quality of the current patch (i.e., the same GUD):
            memorised_patch_intake=expected_quality-current_qualities(current_patch_index);
            %the time the forager expects to stay in the memorised patch (including traveling time):

memorised_patch_time=exploration_times(indices(i))+(memorised_patch_intake*handling_time)+...
                ((1/search_rate)*sum(1./(expected_quality:-1:(current_qualities(current_patch_index)+1))));
            %the mean intake rate expected during this time:
            memorised_patch_intake_rates(i)=memorised_patch_intake/memorised_patch_time;
        end
        %find the best memorised patch to shift into:
        [best_patch_intake_rate i]=max(memorised_patch_intake_rates);
        best_patch_index=indices(i);
    else
        best_patch_intake_rate=0;
    end

    %% calculate the expected intake rate in a new patch
    %the expected patch quality is the number of bites the forager expects to find in a new patch based on its current knowledge of its environment. If this expectation is lower than the current patch quality, the forager will not explore
    expected_patch_quality=round(prior_maximum_patch_quality(b-1));
    if current_qualities(current_patch_index)<expected_patch_quality
        %otherwise, if the forager shifts to a new patch, the current patch density is the GUD and the mean intake rate in the new patch can be calculated based on the assumption that it will have the same GUD.
        new_patch_intake=expected_patch_quality-current_qualities(current_patch_index);
        %the time the forager expects to stay in the new patch (including traveling time):
        new_patch_time=prior_exploration_time(b-1)+(new_patch_intake*handling_time)+...
            ((1/search_rate)*sum(1./(expected_patch_quality:-
1:(current_qualities(current_patch_index)+1))));
        %the mean intake rate expected during this time:
        new_patch_intake_rate=new_patch_intake/new_patch_time;
    else
        new_patch_intake_rate=0;
    end

    %% calculate the expected intake rate in the current patch during the next bite:
```

```
current_patch_intake_rate=(search_rate*current_qualities(current_patch_index))/(1+(search_rate*curr
ent_qualities(current_patch_index)*handling_time));

    %% consume the next bite
    if
(new_patch_intake_rate>=current_patch_intake_rate)&&(new_patch_intake_rate>=best_patch_intake
_rate)
        %in case the forager shifts into a new patch
        patch_exploration(b)=true;
        patch_GUD(b-1)=current_qualities(current_patch_index);
        current_patch_index=max(patch_indices)+1;
        %the new patch maximum patch quality is drawn form a Poisson distribution with a minimum value
of 2 bites.
        current_maximum_patch_quality=poissrnd(mean_maximum_patch_quality-2)+2;
        maximum_patch_qualities=[maximum_patch_qualities current_maximum_patch_quality];
        %the maximum patch quality prior is updated based on the quality of the new patch
        prior_maximum_patch_quality(b)=(theta*current_maximum_patch_quality)+((1-
theta)*prior_maximum_patch_quality(b-1));
        %the new patch exploration time is drawn form an exponential distribution with a minimum value
of 1 bite.
        current_exploration_time=exprnd(mean_exploration_time-1)+1;
        exploration_times=[exploration_times current_exploration_time];
        %the exploration time prior is updated based on the time to the new patch
        prior_exploration_time(b)=(theta*current_exploration_time)+((1-theta)*prior_exploration_time(b-
1));
        %the time required to consume a single bite is the inverse of the type II functional response + the
time spent traveling:

time(b)=(1/(search_rate*current_maximum_patch_quality))+handling_time+current_exploration_time;
        %update the patch qualities vector:
        current_qualities=[current_qualities current_maximum_patch_quality-1];

    elseif
(best_patch_intake_rate>current_patch_intake_rate)&&(best_patch_intake_rate>new_patch_intake_ra
te)
        %in case the forager shifts into the best memorised patch.
        patch_GUD(b-1)=current_qualities(current_patch_index);
        current_patch_index=best_patch_index;
        %in the absence of new information, the priors remain unchanged.
        prior_maximum_patch_quality(b)=prior_maximum_patch_quality(b-1);
        prior_exploration_time(b)=prior_exploration_time(b-1);
```

```
        %the time required to consume a single bite is the inverse of the type II functional response + the time spent traveling:

time(b)=exploration_times(current_patch_index)+(1/(search_rate*round(current_qualities(current_patch_index))))+handling_time;
        %update the patch qualities vector:
        current_qualities(current_patch_index)=round(current_qualities(current_patch_index))-1;

    else
        %in case the forager stays in the current patch.
        %in the absence of new information, the priors remain unchanged.
        prior_maximum_patch_quality(b)=prior_maximum_patch_quality(b-1);
        prior_exploration_time(b)=prior_exploration_time(b-1);
        %the time required to consume a single bite is the inverse of the type II functional response:
        time(b)=(1/(search_rate*current_qualities(current_patch_index)))+handling_time;
        %the current patch quality is updated after the consumption of a single bite
        current_qualities(current_patch_index)=current_qualities(current_patch_index)-1;
    end
    %repeat until all bites are consumed
end
```
